# Comprehensive Genomic Profiling (CGP)-Informed Personalized Molecular Residual Disease (MRD) Detection: An Exploratory Analysis from the PREDATOR Study of Metastatic Colorectal Cancer (mCRC) Patients Undergoing Surgical Resection

**DOI:** 10.3390/ijms231911529

**Published:** 2022-09-29

**Authors:** Sara Lonardi, Halla Nimeiri, Chang Xu, Daniel R. Zollinger, Russell W. Madison, Alexander D. Fine, Ole Gjoerup, Cosimo Rasola, Valentina Angerilli, Shruti Sharma, Hsin-Ta Wu, Charuta C. Palsuledesai, Meenakshi Malhotra, Alexey Aleshin, Fotios Loupakis, Elise Renkonen, Priti Hegde, Matteo Fassan

**Affiliations:** 1 Veneto Institute of Oncology IOV-IRCCS, via Gattamelata 64, 35128 Padua, Italy; 2 Foundation Medicine Inc., 150 Second Street, Cambridge, MA 02141, USA; 3 Department of Surgery, Oncology and Gastroenterology, University of Padua, via 8 Febbraio, 2-35122 Padua, Italy; 4 Department of Medicine (DIMED), Surgical Pathology Unit, University of Padua, via Giustiniani, 35128 Padua, Italy; 5 Natera Inc., 13011A McCallen Pass, Austin, TX 78753, USA

**Keywords:** circulating tumor DNA (ctDNA), metastatic colorectal cancer (mCRC), molecular residual disease (MRD), carcinoembryonic antigen (CEA), comprehensive genomic profiling (CGP)

## Abstract

A majority of patients with metastatic colorectal cancer (mCRC) experience recurrence post curative-intent surgery. The addition of adjuvant chemotherapy has shown to provide limited survival benefits when applied to all patients. Therefore, a biomarker to assess molecular residual disease (MRD) accurately and guide treatment selection is highly desirable for high-risk patients. This feasibility study evaluated the prognostic value of a tissue comprehensive genomic profiling (CGP)-informed, personalized circulating tumor DNA (ctDNA) assay (FoundationOne^®^Tracker) (Foundation Medicine, Inc., Cambridge, MA, USA) by correlating MRD status with clinical outcomes. ctDNA analysis was performed retrospectively on plasma samples from 69 patients with resected mCRC obtained at the MRD and the follow-up time point. Tissue CGP identified potentially actionable alterations in 54% (37/69) of patients. MRD-positivity was significantly associated with lower disease-free survival (DFS) (HR: 4.97, 95% CI: 2.67–9.24, *p* < 0.0001) and overall survival (OS) (HR: 27.05, 95% CI: 3.60–203.46, *p* < 0.0001). Similarly, ctDNA positive status at the follow-up time point correlated with a marked reduction in DFS (HR: 8.78, 95% CI: 3.59–21.49, *p* < 0.0001) and OS (HR: 20.06, 95% CI: 2.51–160.25, *p* < 0.0001). The overall sensitivity and specificity at the follow-up time point were 69% and 100%, respectively. Our results indicate that MRD detection using the tissue CGP-informed ctDNA assay is prognostic of survival outcomes in patients with resected mCRC. The concurrent MRD detection and identification of actionable alterations has the potential to guide perioperative clinical decision-making.

## 1. Introduction

Metastatic colorectal cancer (mCRC) is the most advanced stage of CRC, and is associated with the poorest outcomes, with a five-year survival rate of only 14% [1,2]. Curative-intent surgery is an important option for patients with oligometastatic CRC. Where technically feasible, some patients can achieve long-term survival benefits or even cure post-resection. However, the majority of patients (60–70%) will relapse [3]. In the postoperative setting, current National Comprehensive Cancer Network (NCCN) guidelines recommend either observation or an active systemic therapy regimen for a maximum of 6 months, a choice historically based on clinical and pathological risk factors. The choice of regimen depends on several factors, such as prior chemotherapy treatment, the type of tumor (synchronous or metachronous), the response rates to neoadjuvant therapy, and associated safety issues [4]. For resectable synchronous liver and/or lung metastasis, as well as resectable metachronous disease, NCCN guidelines recommend a preferred treatment course of resection followed by adjuvant FOLFOX or CAPEOX [4]. Capecitabine or 5-FU with leucovorin are also recommended as alternative adjuvant treatments. Recently, immunotherapy (IO) without resection was added to NCCN guidelines as a recommended alternative therapeutic approach in mCRC patients with high levels of microsatellite instability (MSI-H), but limited evidence of IO in the post-resection setting exists [4].

Although a number of studies have demonstrated the short-term benefits of adding adjuvant systemic therapy to surgery, they failed to establish a benefit in overall survival [5,6,7,8,9]. To this end, a diagnostic test that can accurately assess molecular residual disease (MRD) status post-resection and can guide treatment selection in MRD-positive patients based on tumor-specific alterations is highly desirable. Traditionally, carcinoembryonic antigen (CEA) has been the most used blood-based biomarker for CRC patients to assess disease status in the post-surgical surveillance setting. However, several studies have reported its limited clinical utility, with a sensitivity of predicting recurrence between 50–80%, and a high rate of false positives and negatives [10,11,12,13]. MRD assessment using circulating tumor DNA (ctDNA) has emerged as an important biomarker and detection of ctDNA has been shown to be associated with poorer prognosis in patients with CRC [14,15,16,17,18,19].

Here, we sought to demonstrate the validity of a tissue comprehensive genomic profiling (CGP)-informed ctDNA assay, FoundationOne^®^Tracker, for MRD detection and to determine its prognostic value in patients with mCRC who underwent curative-intent surgery. The FoundationOne Tracker assay is designed to track clinically actionable mutations in the MRD setting that can further guide treatment selection in patients. Furthermore, FoundationOne Tracker does not require germline sampling, which sometimes is not feasible or practical, and is based on tissue profiling with the widely available FoundationOne^®^CDx assay.

## 2. Results

**Figure 1:**
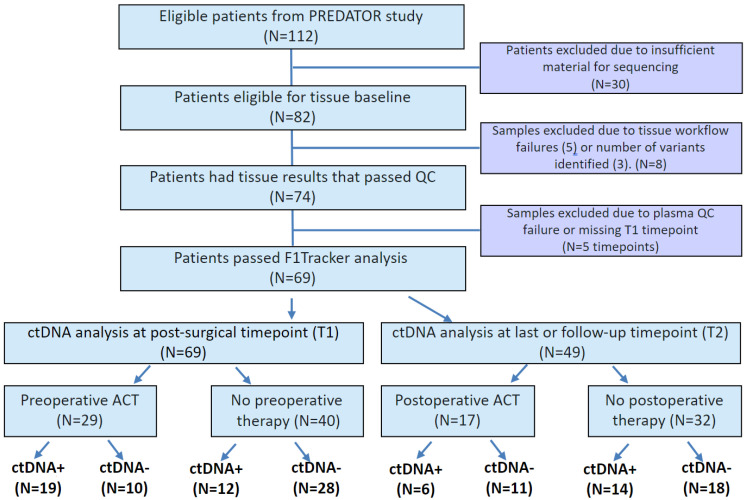
Consort Diagram of patients monitored with FoundationOne^®^Tracker. PREDATOR study and full patient population are described previously [14]. ACT = adjuvant chemotherapy.

In this study, tissue samples were available for 82 patients with median age 60.1 years. Of these, three patients were excluded due to detection of <2 monitorable, somatic variants in the tumor tissue, and five patients were excluded due to tissue workflow failure. In the remaining 74 patients, 69 (93%) passed plasma QC and had T1 plasma time point available for ctDNA analysis (Figure 1). Of these 69 patients, 49 patients had plasma samples available at the time of radiologic evidence of progressive disease or last follow-up (time point T2). Patient characteristics and demographics are detailed in Table 1. Of the 69 patients analyzed, 35 (50.7%) presented with synchronous tumors and the remaining (49.3%) with metachronous tumors. Twenty-nine (42%) patients received preoperative treatment and 27 (39.1%) patients received postoperative treatment. CEA status was available for 28 patients, of whom CEA-positivity was observed in 23 (82.1%) patients preoperatively and in 13 (46.4%) patients postoperatively. Additionally, actionable variants were identified in resected tumors.

### 2.1. FoundationOne Tracker Measures Cancer-Associated Alterations across a Large Range of Variant Allele Frequencies

**Figure 2:**
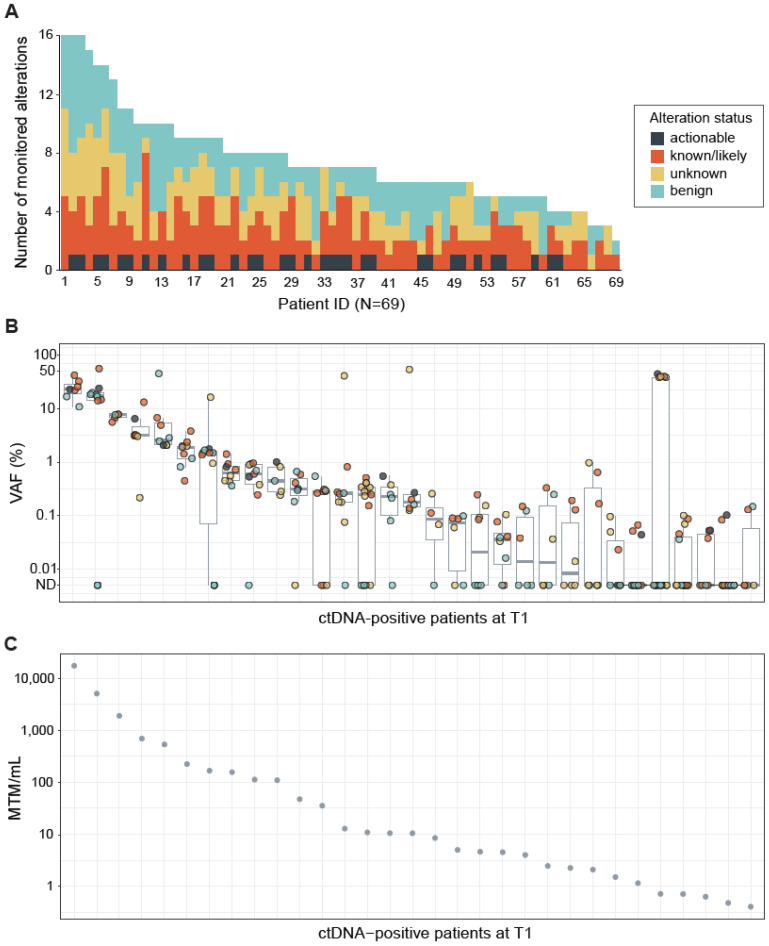
(**A**) Bar plot showing the variants designed for each sample and variant status. Actionable and known/likely are cancer-associated alterations. Benign alterations are intronic or synonymous alterations. Median monitorable alterations per sample = 7. (**B**) Variant Allele Frequency (VAF) of each monitored alteration in ctDNA-positive samples at the postsurgical time point (T1). Boxplot indicates the distribution of VAF values. Average VAF of all detected alterations = 6.0%, range = 0.014 to 56.5%. Alteration status is represented by color. (**C**) Assessment of MTM/mL for each ctDNA-positive patient. ND = Not detected, unknown = variants of unknown significance.

CGP performed on the tumor tissue DNA identified a median of seven (range 2–16) alterations per sample. Actionable alterations were identified in 37 (54%) of 69 resected tumor tissues (Figure 2A). All of the actionable variants identified were RAS-activating mutations (*KRAS*, N = 36; *NRAS*, N = 1) (Table 1). Furthermore, known or likely pathogenic alterations were identified in 68 out of 69 (99%) patients (Figure 2A). Variants of unknown significance in cancer-associated genes were detected in 57 (83%) patients and benign variants (intronic or synonymous mutations) were detected in 63 (91%) patients (Figure 2A). Alterations detected by the tissue CGP-informed ctDNA assay were distributed across a wide ranges of variant allele frequencies (VAFs) (mean VAF = 6.0%, range 0.014–56.5%; Figure 2B) and mean tumor molecules per mL (MTM/mL values) (mean MTM/mL = 848.9, range 0.406–17,189.1; Figure 2C) at T1. The observed VAFs were independent of variant status (actionable, known/likely cancer-associated, unknown, or benign) (Figure 2B).

### 2.2. Landscape of Monitored and Non-Monitored Alterations

**Figure 3:**
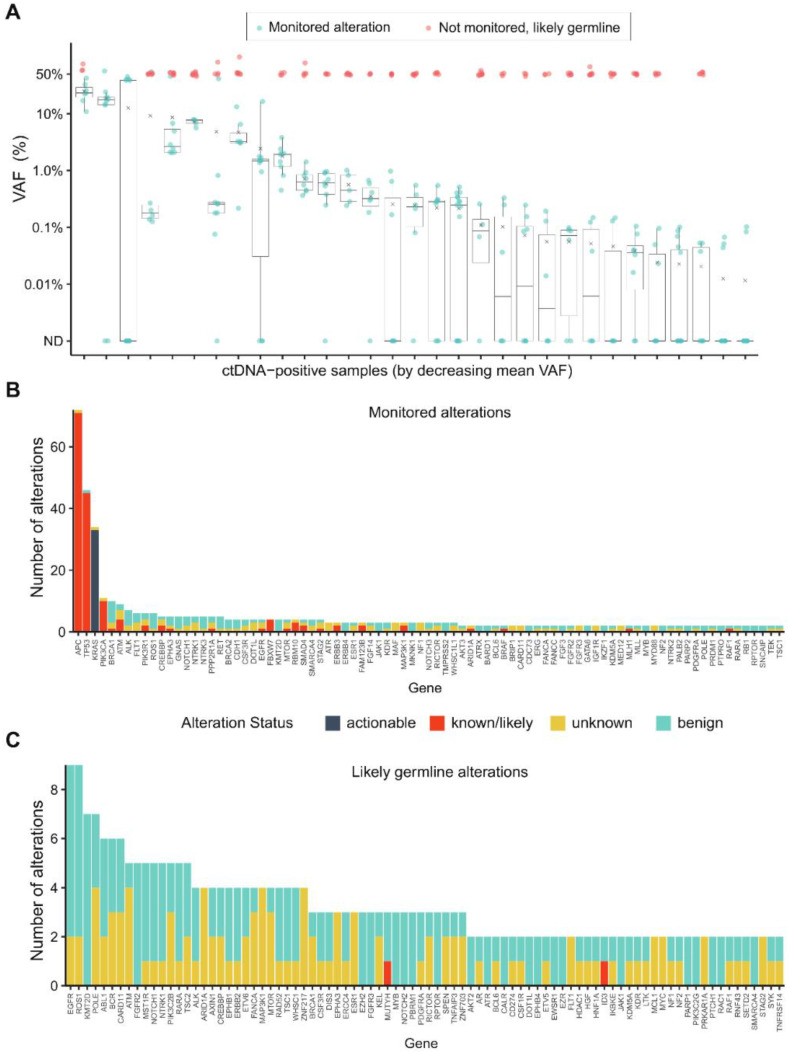
(**A**) Variant Allele Frequency (VAF) of each monitored alteration in ctDNA-positive samples at the postsurgical time point (T1) showing monitored variants (blue points) for each sample, as well as non-monitored variants with VAF ≥ 45% that are likely of germline origin (red points). Boxplots represent the distribution of monitored alterations only. (**B**) Long-tail plot of monitored alterations according to variant status and gene name. (**C**) Long-tail plot of non-monitored alterations of likely germline origin according to variant status and gene name.

Additional analyses were performed to characterize the variants that were monitored versus those not monitored and likely to be of germline origin (Figure 3A). Likely germline variants, defined as those with a VAF ≥ 45% in the ctDNA assay, were filtered out by the algorithm. The frequency of the monitored alterations (Figure 3B) and likely germline alterations (Figure 3C) were assessed by gene. All of the actionable and most of the known/likely variants were included for monitoring. The top five monitored alterations belonged to *APC*, *TP53*, *KRAS*, *PIK3CA,* and *BRCA1,* whereas the top five likely germline variants were detected in *EGFR*, *ROS1*, *KMT2D*, *POLE,* and *ABL1*. The majority of germline alterations were unknown or benign, with the exception of one alteration each in familial cancer genes *MUTYH*, *CHEK2*, and *FANCL*, as well as one germline alteration in *ID3*. Of the monitored alterations, 88.8% were single nucleotide variants (SNVs) and 11.2% were indels; whereas 87.1% of the non-monitored alterations of likely germline origin were SNVs and 12.9% were indels. Next, we evaluated whether the monitored alterations were recurrent by assessing 14 patients that were ctDNA positive at both T1 and T2 time points. Of the total 214 variant calls made for these 14 patients, 88.8% (190/214) were the same between T1 and T2, whereas 11.2% (24/214) changed from T1 to T2.

### 2.3. ctDNA Detection at Postsurgical Time Point Is Predictive of DFS and OS

**Figure 4:**
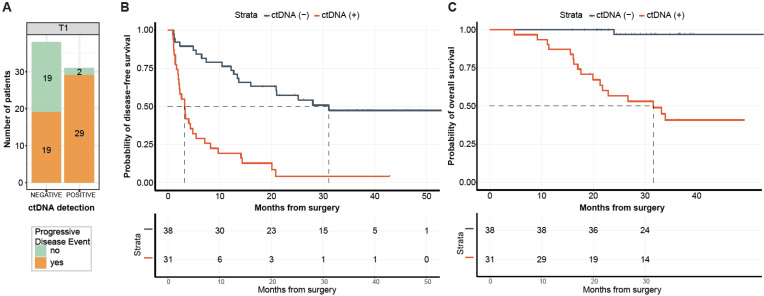
(**A**) ctDNA detection rates at time point T1. (**B**,**C**) Kaplan–Meier estimates for 69 patients monitored by FoundationOne^®^Tracker stratified by ctDNA detection (MRD) at the postsurgical time point (T1). Median lead time = 2.4 months, based on 29 patients with ctDNA (+) at T1 and disease progression. DFS = Disease-free survival, defined as time from metastases resection to the date of the first evidence of progressive disease, as defined by RECIST criteria. OS = overall survival.

**Figure 5:**
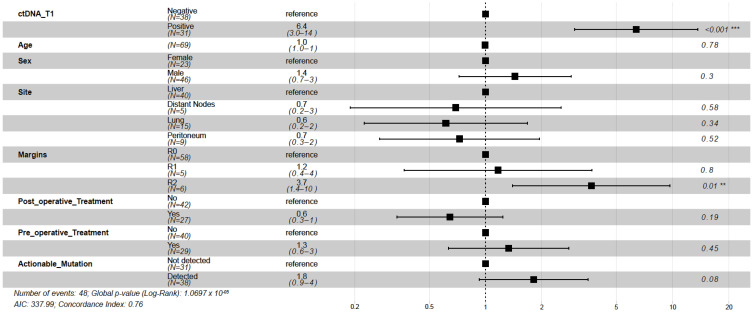
Forest plot depicting the multivariate analysis of prognostic factors and their association with DFS, as indicated by the Hazard Ratio. R2 resection margin had a significant association with disease-free survival (*p* = 0.008). ctDNA detection at the postsurgical time point (T1) was the most significant prognostic factor (*p <* 0.001). ** represents *p* < 0.01, *** represents *p* < 0.001.

Of the 69 patients with post-surgical time point (T1) available prior to adjuvant chemotherapy (ACT) (median: 26.5 days; range: 8–99.5 days), 31 (44.9%) patients were MRD-positive (defined as ctDNA-positive at T1) and 38 (55.1%) were MRD-negative (ctDNA-negative at T1, Figure 4A). Of the 31 MRD-positive patients, 29 (PPV = 93.5%) eventually experienced disease progression (Figure 4A) with a median lead time of 2.4 months (range: -0.083-19.73 months). One of the two ctDNA-positive patients that did not progress received ACT. In comparison with MRD-negative patients, patients with MRD-positivity exhibited an inferior median DFS of 3.2 months vs. 31.1 months, respectively, and were five times more likely to progress (HR: 4.97, 95% CI: 2.67–9.24, *p <* 0.0001; Figure 4B). Similarly, MRD-positive status was significantly associated with lower OS (HR: 27.05, 95% CI: 3.60–203.46, *p <* 0.0001; Figure 4C). At the end of the follow-up, 97% (37/38) of MRD-negative patients were alive compared with 45% (14/31) of MRD-positive patients. Overall, at the MRD (T1) time point, the tissue CGP-informed ctDNA assay demonstrated a patient level sensitivity of 60.4% (29 MRD-positive patients out of 48 patients with disease progression), a specificity of 90.4% (19 MRD-negative patients out of 21 non-progression patients). These results indicate that ctDNA is a strong prognostic biomarker of DFS and OS in post-surgery patients with mCRC. In the multivariate analysis, ctDNA-based MRD status at T1 was the most significant prognostic factor associated with DFS (HR: 6.39, 95% CI: 3.00–13.60, *p <* 0.001; Figure 5) along with the R2 resection margin (HR: 3.67, 95% CI: 1.40–9.70, *p* = 0.008; Figure 5) when compared with other clinicopathologic factors.

### 2.4. ctDNA Detection at Follow-Up Time Point Is Predictive of DFS and OS

**Figure 6:**
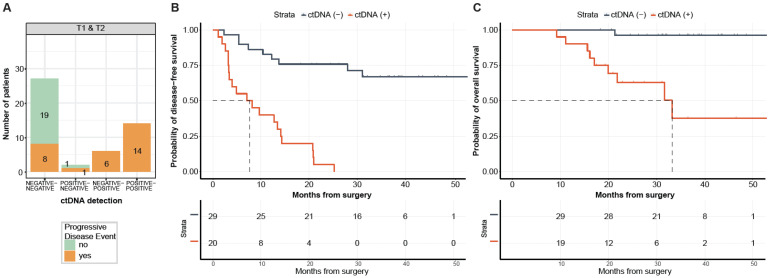
(**A**) ctDNA detection rates across two time points (T1 + T2). (**B**,**C**) Kaplan–Meier estimates for 49 patients monitored by FoundationOne^®^Tracker across two time points (T1 + T2) stratified by ctDNA detection at the last or follow-up time point (T2).

A total of 49 patients had plasma samples available at both T1 and T2. To determine the correlation of serial ctDNA detection with DFS and OS, patients were stratified by their ctDNA status at T2 time point. Of the 49 patients, 20 were ctDNA-positive (14 remained and 6 turned positive from T1), all of whom progressed (PPV = 100%). Of the 29 ctDNA-negative patients, 27 consistently stayed negative, however, of these only eight progressed (Figure 6A). Among the two patients that became positive from negative, one patient progressed, despite receiving ACT. The overall sensitivity and specificity at T2 were observed to be 69% and 100%, respectively. As shown in Figure 6B,C, ctDNA positive status at the follow-up time point T2 correlated with a marked reduction in DFS (HR: 8.78, 95% CI: 3.59–21.49, *p <* 0.0001) and OS (HR: 20.06, 95% CI: 2.51–160.25, *p <* 0.0001).

### 2.5. Comparison of ctDNA with CEA and Their Correlation with Disease Progression

**Figure 7:**
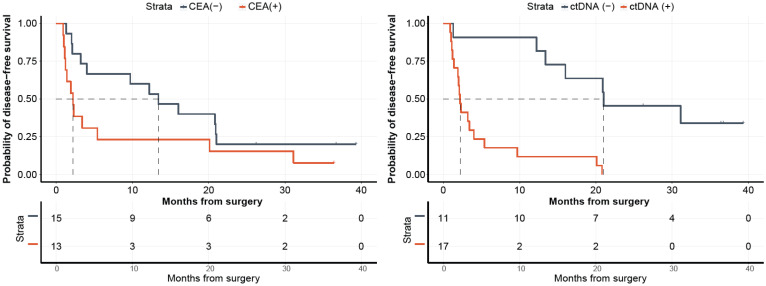
Kaplan–Meier estimates for 28 patients with Carcinoembryonic antigen (CEA) test results and ctDNA monitoring. The population when stratified by CEA result did not show a significant association with DFS (left). In contrast, patients stratified by ctDNA detection (MRD) at the postsurgical time point (T1) did show a significant association with DFS (right; *p* = 0.0001).

For a subset of patients (N = 28), with both post-surgical ctDNA and CEA results available, we analyzed their correlation with disease progression. As observed in Figure 7, while patients stratified by ctDNA status showed a significant association with DFS with ctDNA-positivity being highly predictive of disease progression (HR: 7.95, 95% CI: 2.54–24.89, *p <* 0.0001), CEA status was not observed to be predictive of DFS (HR: 1.97, 95% CI: 0.88–4.43, *p* = 0.0945).

## 3. Discussion

This study demonstrates the feasibility of a tissue CGP-informed personalized ctDNA assay for MRD detection in patients with mCRC, identifying a population with inferior DFS (HR: 4.97; *p <* 0.0001; Figure 4B) and OS (HR: 27.05; *p <* 0.0001; Figure 4C) that might benefit from future adjuvant therapy. The prognostic value of ctDNA-based MRD detection in patients with mCRC (stage IV) has previously been demonstrated [14,20]. A recent meta-analysis of 28 studies analyzing a total of 2823 patients examined the correlation between the clinical outcomes of stage IV mCRC patients with ctDNA status. This study showed the association of ctDNA-positivity at post-definitive treatment (surgery or chemotherapy) with poorer survival outcomes (OS: HR 2.2, *p <* 0.00001; PFS: HR 3.15, *p <* 0.0000) [20]. Our assay demonstrated patient level sensitivity and specificity of 60.4% and 90.4%, respectively, at the T1 MRD time point with a PPV of 93.5%. The performance of the assay substantially improved with the analysis of serial (combination of two) time points exhibiting a PPV of 100%.

Increasing the understanding of clinically actionable alterations can reshape the treatment paradigm in mCRC [21]. In this study, the tissue CGP-informed ctDNA assay detected actionable variants in 54% (37/69) of the patients and other known/likely cancer-associated variants in most of the patients. Actionable alterations were identified in *KRAS* (36/37, 97.3%) and *NRAS* (1/37, 2.7%). Previous studies have shown alterations in KRAS to be a negative predictor of treatment response (targeted EGFR therapy) and thus identifying these actionable mutations can provide clinically important information in the management of patients with mCRC [22,23]. The clinical value of the FoundationOne Tracker assay is intended for concurrent identification of actionable and clinically relevant genomic mutations in mCRC as well as MRD status, which will enable clinicians to ultimately personalize and adapt therapy to improve outcomes. Additionally, the assay showed a more significant association with DFS than CEA. CEA is traditionally known to be a less sensitive and unreliable biomarker as its levels can be influenced by factors outside of tumor growth, such as chemotherapy treatment [24]. Additionally, CEA levels are not always detectable, especially in tumors that do not secrete high levels of CEA, such as MSI-high CRC [25,26,27].

This study also characterized the landscape of monitored versus germline alterations (Figure 3). The monitored alterations were actionable or known/ likely variants predominantly mapping to *APC*, *TP53*, *KRAS,* and *PIK3CA*, which are genes frequently altered in mCRC. Conversely, there was little difference in frequency among the likely germline alterations, and the variant status was mainly unknown or benign.

While this study establishes the feasibility of a tissue CGP-informed ctDNA assay, it is associated with some limitations. The retrospective and non-interventional study design and the use of archived specimens resulted in the exclusion of some samples due to insufficient material for CGP. Additionally, some patient samples had prior systemic treatment, which resulted in necrotic tissue specimens. However, despite the reduced sample size, our study was able to validate the prognostic value of tissue CGP-informed personalized ctDNA assay. We anticipate that in prospective studies, the availability of fresh patient samples will lead to fewer exclusions and lower failure rates. Other limitations include ctDNA monitoring performed at only two time points, wherein additional time points may increase the prognostic value of ctDNA. The sensitivity of the F1 Tracker at the T1 time point was observed to be comparable to the previously reported Signatera assay and consistent with sensitivities reported for multiple ctDNA MRD studies [28]. The FoundationOne Tracker utilizes a CGP-informed approach, whereas the Signatera assay utilizes a whole exome sequencing (WES)-based approach, which could lead to the differences in the number of variants tracked. Our study, however, was not scoped for any comparison and was restricted to demonstrating the feasibility of performing ctDNA testing using a CGP-based panel, highlighting the main benefit of not needing germline sampling. Finally, while many previous studies have included the sequencing of germline variants from buffy coat, we demonstrate that an algorithm can efficiently filter out germline alleles, but we cannot exclude the possibility that some germline variants remain despite filtering. However, this approach can be beneficial in certain scenarios where the source specimen is not available for germline sequencing. Overall, the study demonstrates the advantage of a CGP-based ctDNA assay, which includes the identification of actionable alterations, which, along with MRD detection, can have the potential to guide perioperative clinical decision-making.

Taken together, the results in the present study indicate the feasibility of using tissue CGP-informed ctDNA assay for MRD detection in patients with mCRC. Postoperative ctDNA-positivity at MRD or at a follow-up time point was associated with poor survival outcomes. Given the potential of MRD-based risk stratification, future prospective studies would be needed to determine the benefit of optimal treatment strategy based on the clinically actionable mutations, followed by treatment response monitoring.

## 4. Materials and Methods

Patients with mCRC enrolled in the PREDATOR study conducted at Instituto Oncologico Veneto, IRCCS, Padua, Italy, in collaboration with the Department of Medicine, University of Padua, Italy, were eligible for inclusion in this study analysis. The PREDATOR study collected informed consent from all patients for participation in the study and was granted Ethics Approval by Local Authorities and was conducted in accordance with the Declaration of Helsinki (CESC Istituto Oncologico Veneto ref no. 2018/66). Tumor tissue samples were available from 82 patients for CGP. ctDNA analysis was performed retrospectively on plasma samples obtained at pre-specified time points. The patient cohort used in this study is similar to a previously published article by Loupakis et al. [14] Patient characteristics, including clinical, pathological, and treatment regimens are presented in Table 1.

### 4.1. Tissue CGP-informed ctDNA assay

The FoundationOne Tracker is a tissue-informed personalized ctDNA monitoring assay for determining molecular and therapeutic response in patients across tumor types. In this study, genomic DNA from resected tumor tissue was collected and extracted as described previously [14]. The CGP of tumor DNA was performed retrospectively using a method adapted from the study by Milbury et al. to identify patient-specific alterations [29]. Briefly, the DNA was extracted from FFPE archival patient samples and was end-repaired, A-tailed, and adapters were ligated, followed by hybrid capture-based next-generation sequencing (NGS) on the Illumina^®^ HiSeq 4000 (Illumina, Inc., San Diego, CA, USA). A proprietary algorithm (Foundation Medicine Inc) was used to select short variants for primer design and exclude non-tumor derived variants (germline, clonal hematopoiesis derived, sequencing artifacts). A novel logistic regression model was implemented to predict the probability of a variant being somatic (somatic probability score) based on the difference between the observed variant allele frequency and the inferred expected germline variant allele frequency. This algorithm directly infers the expected germline allele frequency from known germline single nucleotide polymorphisms (SNPs) located on the adjacent genomic region expected to have the same copy number with the variant in question. The algorithm then filters variants based on the somatic probability score, allele frequency and annotation, and compares the variants with databases of known SNPs and clonal hematopoiesis variants. This approach can select coding non-silent alterations in cancer-associated genes (termed known/likely for alterations with known or likely oncogenic significance or termed unknown for alterations with unknown significance) as well as intronic or synonymous alterations (termed benign) for monitoring.

### 4.2. Variant Selection and Primer Design

To build the tumor-specific ctDNA assay, up to 16 clonal SNVs from CGP results were selected using a proprietary algorithm (Natera, Inc.) with an aim to maximize the detectability of tumor DNA in patients’ plasma. The selected SNVs were used to design PCR amplicons based on optimized design parameters, ensuring the uniqueness of the amplicon sequences in the human genome and the efficiency and compatibility of the amplicons.

### 4.3. Cell-Free DNA Extraction, Library Preparation, and Plasma Multiplex-PCR Next Generation Sequencing Workflow

FoundationOne Tracker was performed retrospectively on cfDNA extracted from 10 mL plasma. Each cfDNA sample was quantified by Quant-iT High Sensitivity dsDNA Assay Kit (Invitrogen) following the manufacturer’s instructions. Up to 66 ng (20,000 genome equivalents) of cfDNA from each plasma sample was used as input into library preparation. The cfDNA was end-repaired, A-tailed, and ligated with custom adapters, as previously described [30]. The purified ligation product was amplified and purified using Ampure XP beads (Agencourt/Beckman Coulter). An aliquot of each library was used as the input into the patient-specific 16-plex PCR reaction. Samples were amplified using the patient-specific assay and barcoded, followed by pooling the amplicons. Sequencing was performed on an Illumina HiSeq 2500 Rapid Run with 50 cycles of paired-end reads using the Illumina Paired End v2 kit. All paired-end reads were merged using Pear software. Bases that do not match in forward and reverse reads or that have a low-quality score were filtered out to exclude sequencing errors. Merged reads were mapped to the hg19 reference genome with Novoalign (http://www.novocraft.com/, accessed on 22 November 2021). Mapped sequencing reads went through a QC process to filter reads that are not on-target PCR products. After the sequencing of the PCR products, the number of reads for each amplicon of a patient-specific assay were determined. Individual targets have an average read depth of >105,000×. Targets with more than 5000× sequencing coverage are included in the analyses.

### 4.4. Plasma Variant Calling

Based on the proprietary error model a confidence score was calculated for each target variant detected using mutant and reference alleles depth of read, as previously described [31]. The presence of tumor DNA in the plasma was determined based on a validated combined confidence that takes all patient-specific variants of the assay into account. In order to make a ctDNA positive call, it is critical to observe at least two SNVs above the selected confidence threshold [31].

### 4.5. Analysis of Monitored and Non-Monitored Variants

To determine the frequency of genes with germline alterations in the CGP tissue assay, primers were designed and added to the mPCR assay for short variants excluded by the FoundationeOne Tracker variant selection algorithm. Likely germline alterations were defined as those excluded variants with VAF ≥ 45% at the enrollment timepoint in ctDNA positive samples.

### 4.6. Statistical Analysis

The ctDNA statistical analysis plan was developed and implemented prior to unblinding of the clinical data. The data assessors were blinded to sample order and patient outcome. The primary outcome measure, DFS, was assessed between the date of metastases resection and the date of the first evidence of progressive disease, as defined by RECIST criteria [32]. The Kaplan–Meier Estimator was used for estimating the survival distributions. Log-rank test was used for comparing two survival distributions with *p* ≤ 0.05 being considered significant. Univariable and Multivariable Cox proportional hazards models were used for estimating the hazard ratio (HR). The association of ctDNA status with DFS was assessed using a univariable Cox proportional hazards model and then using a multivariable Cox proportional hazards model to adjust for prognostic factors. Statistical analyses were carried out in R-4.0.2 using packages survminer, survival, and coxph [33].

## Figures and Tables

**Table 1:** Patient demographics and baseline clinical characteristics. Abbreviations: FP = fluoropyrimidine; CEA = carcinoembryonic antigen; ctDNA = circulating tumor DNA.

Patient characteristics (All patients, N = 69)	N	%
**Sex** Male Female	46 23	66.7 33.3
**Presentation of metastasis** Synchronous Metachronous	35 34	50.7 49.3
**Median age at first diagnosis, median (range), years**	59.5	20.8–82.8
**Median age at diagnosis of metastatic disease, median (range), years**	60.1	22.1–83.3
**Adjuvant therapy administered to metachronous**	26	76.5
**Site of surgery** Liver Lung Peritoneum Other	40 15 9 5	58 21.7 13 7.2
**Presurgical treatment** Doublet with or w/o biologic, FP monotherapy with or w/o biologic, and triplet with or w/o biologic	29	42
**Postsurgical treatment** Doublet with or w/o biologic, FP monotherapy, and triplet with or w/o biologic	27	39.1
**Event** Progressive disease	48	69.6
**CEA status: preoperative (N = 28)** CEA-positive	23	33.3
**CEA status: postoperative (N = 28)** CEA-positive	13	18.8
**Resection margins** R0 R1 R2	58 5 6	84.1 7.2 8.7
**Actionable alterations identified in resected tumor (KRAS = 36, NRAS = 1)**	37	54
**ctDNA detected at first timepoint (T1)**	31	45
**ctDNA detection at last or follow-up timepoint (T2) (N = 49)**	20	41

## Data Availability

All data generated or analyzed during this study are included in this article. Further inquiries can be directed to the corresponding author.

## Abbreviations

mCRC	Metastatic colorectal cancer
MRD	Molecular residual disease
CGP	Comprehensive genomic profiling
ctDNA	Circulating tumor DNA
HR	Hazard ratio
DFS	Disease-free survival
OS	Overall survival
CEA	Carcinoembryonic antigen
FP	Fluoropyrimidine
NCCN	National Comprehensive Cancer Network
5-FU	5-Fluorouracil
IO	Immunotherapy
MSI-H	Microsatellite instability high
QC	Quality control
ACT	Adjuvant chemotherapy
VAF	Variant allele frequency
MTM/mL	Mean tumor molecules per milliliter
ND	Not detected
PPV	Positive predictive value
CI	Confidence interval
NGS	Next generation sequencing
FFPE	Formalin-fixed, paraffin-embedded
SNP	Single nucleotide polymorphism
SNV	Single nucleotide variant
WES	Whole exome sequencing
